# Super-Transparent Soil for In Situ Observation of Root Phenotypes

**DOI:** 10.3390/molecules29112677

**Published:** 2024-06-05

**Authors:** Jinchun Xie, Qiye Wu, Liping Feng, Junfu Li, Yingjie Zhou, Guo-Zhang Wu, Yongjun Men

**Affiliations:** 1 State Key Laboratory for Modification of Chemical Fibers and Polymer Materials, College of Materials Science and Engineering, Donghua University, Shanghai 201620, China; xjc_6701xl@163.com (J.X.); wuqiye571@163.com (Q.W.); lijunfu2022@126.com (J.L.); zhouyj@dhu.edu.cn (Y.Z.); 2 Shanghai Collaborative Innovation Center of Agri-Seeds, Joint Center for Single Cell Biology, School of Agriculture and Biology, Shanghai Jiao Tong University, 800 Dongchuan Road, Shanghai 200240, China; lipingfeng@sjtu.edu.cn

**Keywords:** transparent soil, plant culture media, root phenotypes observation

## Abstract

Transparent soil (TS) presents immense potential for root phenotyping due to its ability to facilitate high-resolution imaging. However, challenges related to transparency, mechanical properties, and cost hinder its development. Herein, we introduce super-transparent soil (s-TS) prepared via the droplet method using low acyl gellan gum and hydroxyethyl cellulose crosslinked with magnesium ions. The refractive index of the hydroxyethyl cellulose solution (1.345) closely aligns with that of water (1.333) and the low acyl gellan gum solution (1.340), thereby significantly enhancing the transmittance of hydrogel-based transparent soil. Optimal transmittance (98.45%) is achieved with polymer concentrations ranging from 0.8 to 1.6 wt.% and ion concentrations between 0.01 and 0.09 mol·L^−1^. After 60 days of plant cultivation, s-TS maintains a transmittance exceeding 89.5%, enabling the detailed visualization of root growth dynamics. Furthermore, s-TS exhibits remarkable mechanical properties, withstanding a maximum compressive stress of 477 kPa and supporting a maximum load-bearing depth of 186 cm. This innovative approach holds promising implications for advanced root phenotyping studies, fostering the investigation of root heterogeneity and the development of selective expression under controlled conditions.

## 1. Introduction

Most crop breeding research is primarily focused on root phenotype, as it is crucial for achieving high crop yields and efficient nutrient absorption [1,2]. Root trait breeding is emerging as the forefront of next-generation breeding research, aided by the development of high-throughput phenotyping platforms and molecular markers [3,4,5]. Phenotyping methods play a critical role in the discovery of beneficial root traits, which can be integrated into future varieties, and in the identification of marker–trait associations and candidate genes for marker-assisted selection [4,6]. However, the observation of plant root phenotypes faces significant limitations due to the nature of the substrate [7]. Field soils are largely opaque to most forms of radiation and exhibit limited control over the heterogeneities that influence root development, such as water availability gradients, nutrient concentrations, mechanical properties, and porosity [8]. Conversely, transparent culture methods (e.g., hydroponics, aeroponics, and gels) do not facilitate the observation of field-relevant phenotypes and growth conditions [9]. Therefore, developing a transparent substrate that closely resembles real soil and establishing a new method for observing plant root growth will greatly advance breeding and seedling research.

Soil is a multifaceted matrix, fundamental to terrestrial life, consisting of minerals, organic particles, and a diverse biological community [10]. It is structured into solid and liquid phases: the solid comprising minerals and organic matter, and the liquid phase containing dissolved minerals and gases [11]. In unsaturated soils, where pores are partially filled with air, macropores rich in air provide essential gases for the metabolic activities of plants and microorganisms [12]. While imaging technology is vital for studying soil biological processes, soil’s inherent opacity poses significant challenges to research observations [13,14]. Current imaging techniques, such as X-ray tomography (CT), nuclear magnetic resonance (MRI), and in situ multispectral imaging systems, are limited by their inability to facilitate real-time imaging or their cumbersome operational requirements, hindering large-scale observation [15,16,17]. Optical imaging systems, although rapidly developing and widely used in cell and tissue studies, have not effectively penetrated root observation in soil due to these limitations. For example, optical projection tomography (OPT) and selective plane illumination microscopy (SPIM) have revolutionized imaging in other fields but are impeded in soil applications by the opaque nature of the substrate [18,19].

Traditional transparent substrates used in plant culture, such as agar and low acyl gellan gum, have inherent shortcomings [20,21]. Agar, derived from red algae, and low acyl gellan gum, a linear polysaccharide polymer, are limited in their ability to mimic nature’s soil environment, affecting the authenticity of observed plant root growth [3,22,23].

Innovations in TS have attempted to address these challenges [24,25]. In 2012, Downie H. et al. from the University of Manchester, UK, inspired by granular materials used in soil physics and fluid dynamics, conducted a study to simulate the particle structure in soil [26]. They designed a matrix composed of Nafion solid particles with a pore network containing liquid and air. Nafion particles, which are based on sulfonated tetrafluoroethylene, are a fluoropolymer resin with a refractive index (RI, 1.368) similar to that of water (1.333), making the soil transparent when mixed with water, leading to the term “transparent soil”. By mimicking the essential physical and chemical properties that support plant and microbial growth, the researchers achieved root growth similar to that observed in natural soil and sand, thereby demonstrating physical heterogeneity in the growth matrix. This approach allowed for imaging at the whole root system level using inexpensive OPT. However, the Nafion material requires chemical treatment before use, does not absorb water or nutrients, contains high concentrations of sorbitol (0–13 wt.%) in the index-matching solution, causing osmotic stress in plants, and is relatively expensive (USD ~1000·per kg), making mass production impractical [27]. Another transparent soil system, developed by Ludovico’s team at Iowa State University in the United States, is a porous medium composed of hydrogel beads filled with nutrient solution [28]. These beads, with controllable size and hardness, contain pores storing oxygen necessary for plant growth. Given the high water content of the hydrogel, the beads have a refractive index similar to that of water, facilitating transparency when filled with the nutrient solution. This system allows for real-time observation of cultivated plants through cameras and microscopes. The hydrogel beads are produced rapidly and inexpensively by dropping a solution of low acyl gellan gum and alginate into a stirred MgCl_2_ solution. Experimental evidence indicates that six of the seven key phenotypes of soybeans, when planted in the transparent gel soil granular medium, were not significantly different from field soil cultivation, surpassing hydroponics, which only exhibited two key phenotypes and presented root defects. Notably, a key gene for oxygen response [non-symbiotic hemoglobin (nsHB); Glyma.11G121800] showed significantly higher expression in hydroponics than in gel beads and real soil, highlighting the variability of plant root phenotypes in gel particles. Although hydroponics offers closer resemblance to natural soil, the sterile environment and the use of sodium alginate prevent the hydrogel system from achieving ultra-high transparency and high capacity, which limits the size of the root observation window and the clarity of imaging [22].

**Figure 1:**
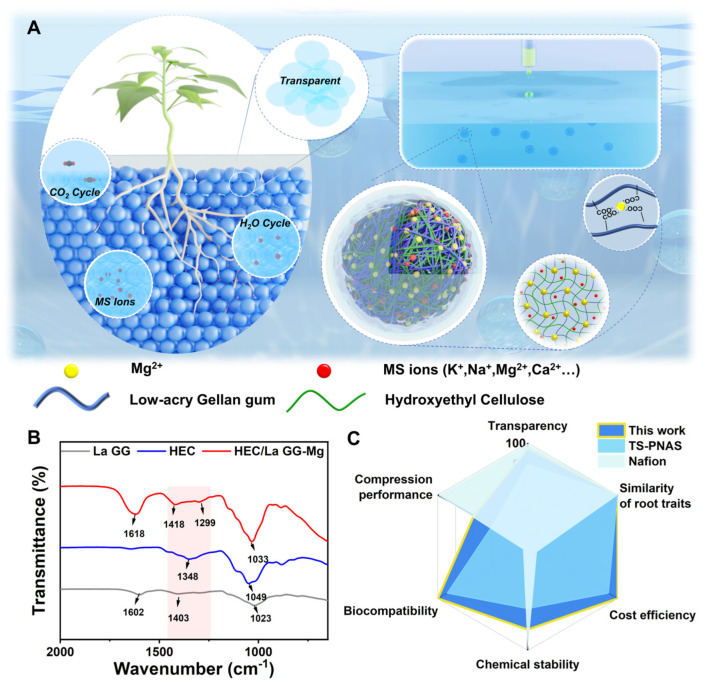
(**A**) Schematic diagram of the synthesis, preparation, and application process of s-TS. (**B**) Infrared spectra of HEC, La GG, and H_4_G_8_ 0.01 Mg hydrogels. (**C**) Performance comparisons of s-TS prepared in this work with other TSs.

In response to these challenges, our study presents a novel approach using hydroxyethyl cellulose (HEC) [29,30], which exhibits very similar RI (1.345) as water (1.333) and MS solution (1.340) [31], to create a natural hydrogel-based super-transparent soil (s-TS). This s-TS allows for plant culture in natural conditions while maintaining 89.5% light transmittance after a two-month culture cycle. Through cultivating rapeseed, we observed significant improvements in biomass and root phenotypes compared to traditional soil and hydroponic systems. Besides transparency, the biocompatibility, cost efficiency, and similarity of root traits of s-TS surpass those of the reported TS systems (Figure 1C). Additionally, our innovative peristaltic pump system enables the automated, large-scale preparation of this transparent soil, paving the way for more accurate, large-scale root observation and study.

## 2. Results and Discussion

Hydrogels-based TSs enable the design of matrices for heterogeneous studies and more suitable artificial substrates for root growth by tuning the structure of polymer networks. We employed a peristaltic pump to extrude numerous droplets, which were immersed in a solution containing abundant magnesium ions, to prepare a large number of s-TS hydrogel beads. The plants to be studied were then transplanted into s-TS, and when the plant roots had grown to a certain stage, root observation and in situ root image analysis were performed, as shown in Figure 1A. The formation mechanism of s-TS hydrogel beads mainly involves the ionic cross-linking reaction between the carboxylate anions of the low acyl gellan gum and magnesium ions. The ion sensitivity of the low acyl gellan gum ensures that, when the droplets come into contact with the ionic solution, a network rapidly forms on the surface of the droplets, thus preserving the droplet shape. This reaction progresses rapidly from the outside inwards, resulting in the formation of hard, transparent gel particles. At the same time, the introduced hydroxyethyl cellulose is used as a thickener to significantly improve the surface tension of the polymer droplets, ensuring that the droplets can rapidly enter the liquid surface without collapsing and retain a good shape. After the droplets enter the liquid surface, the outer surface is rapidly cross-linked and soaked in a precipitation bath for a period of time, waiting for the ions to slowly enter the interior to form hard, transparent gel particles. Subsequently, we transplanted 3-day-old rapeseed seedlings into this TS to conduct experiments on plant root growth observation. The stacking system achieves high visibility, good gas–water ratio, and slow-release supply of ionic nutrients based on the characteristics and shape of the gel. Shown in Figure 1B are the infrared spectra of hydrogels formed by hydroxyethyl cellulose, low acyl gellan gum, and H_4_G_8_ 0.01 Mg (1 × MS). For pure hydroxyethyl cellulose, the peaks at 1348 and 1049 cm^−1^ are assigned to the C-H stretching vibrations. The low acyl gellan gum displayed the asymmetric stretching vibration of −COO at 1602 cm^−1^ and the symmetric stretching vibrations at 1403 and 1023 cm^−1^. After cross-linking of low acyl gellan gum with magnesium ions, two new bands appeared at 1418 and 1299 cm^−1^ for the antisymmetric carboxylate anion stretching, which can be attributed to the interaction between carboxylate anions and magnesium ions, forming a semi-interpenetrating single-network hydrogel [32]. Figure 1C presents the performance comparison of our work with other works in the same field in terms of six aspects: transparency, loading capacity, biocompatibility, chemical stability, root phenotype, and preparation cost. It can be seen that the s-TS prepared in this work has been improved in transparency, biocompatibility, chemical stability, preparation cost, and root phenotype. Although the loading capacity is lower than that of Nafion resin particles, it is significantly higher than that of similar hydrogel-based transparent soils.

**Figure 2:**
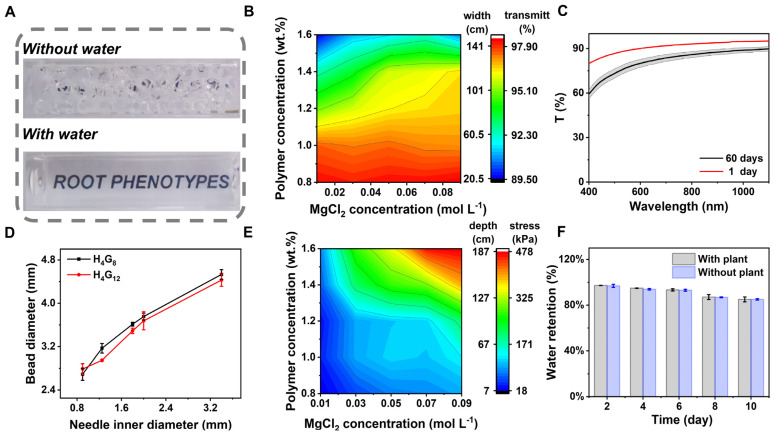
(**A**) Photographs of H_4_G_8_ 0.01 Mg hydrogel beads particle network before (top) and after (bottom) hydration with water. (**B**) Transmittance (at 1080 nm) of s-TS (1 × MS) as a function of polymer and MgCl_2_ concentration. The colored map also shows the optical path length that results in a 10% drop in transmission at 1080 nm. (**C**) Transmittance of H_4_G_8_ 0.01 Mg hydrogel beads before and after colonizing with plants. (**D**) Effect of varying spinneret diameter at two polymer concentrations on hydrogel bead size. (**E**) Collapse stress of s-TS (1 × MS) as a function of polymer and MgCl_2_ concentration. The colored map also shows the maximum hydration depth at which the network is collapsed. (**F**) Moisture content of H_4_G_8_ 0.01 Mg hydrogel beads in a humid environment (99% relative humidity) over 10 days.

When s-TS is stacked, a large number of pores communicating with the atmosphere are formed. Light passing through the stacked system undergoes light refraction at the interface between the hydrogel and the gas, causing a large amount of light refraction loss, making the system significantly opaque. However, s-TS has a refractive index close to that of aqueous solutions. Pores can be filled with water or nutrient solution to greatly reduce the loss caused by light refraction, forming a transparent and visible stacked system that is conducive to the observation of root phenotypes and in situ imaging analysis. As shown in Figure 2A, prepared H_4_G_8_ 0.01 Mg hydrogel beads were placed in a cuvette. The images from top to bottom are photographs of H_4_G_8_ 0.01 Mg hydrogel beads with and without water filling, respectively. It can be observed that the letters at the bottom of the cuvette are clearly visible after the water filling gap, and the interface between the hydrogel and the water is nearly invisible. The transmittance of the stacked system without filling is close to 0, and the transmittance of the system rises up to 90% or more after the filling gap, as shown in Supporting Information Figure S1A. This is mainly because the low acyl gellan gum has little effect on the transmittance of transparent soil, and the refractive index (RI) of the thickener hydroxyethyl cellulose solution is 1.368, which is close to the refractive index of water (1.333), so that the hydrogel itself maintains extremely high transparency. The transmittance of the stacked s-TS and the maximum load carried by the hydrogel particles at the bottom, respectively, limit the maximum imaging width and maximum cumulative height of the basin diameter, which can be regulated by the concentrations of polymer and magnesium ions. In addition to utilizing a flatbed scanner, this cost-effective imaging technique is capable of capturing root phenotypes in hydrogel-based transparent soils with comparable or even superior definition. Therefore, we opted to examine the system’s maximum visualization width at 1080 nm. As illustrated in Figure 2B (Figure S1B), the graph depicts the transmittance distribution of the system as polymer and MgCl_2_ concentrations vary. The color gradient on the right indicates the corresponding maximum visualization width at each transmittance level. Notably, as the concentrations of the polymer (low acyl gellan gum) and MgCl_2_ rise, the system’s transmittance decreases marginally. Furthermore, the transmittance of the stacked system is more susceptible to changes in polymer concentration. Meanwhile, Figure S1C showcases the transmittance and maximum visualization width at the maximum visible wavelength (800 nm). Following the cultivation of plants in H_4_G_8_ 0.01Mg hydrogel beads under standard conditions for 60 days, the light transmittance of these beads was reassessed, as demonstrated in Figure 2C. The light transmittance of the transparent soil remained as high as 94.47% of the initial value after being planted for a long time, indicating stable light transmittance performance. The loss of transmittance may be caused by the effect of rhizosphere microorganisms. Meanwhile, hydrogel beads with various diameters could be prepared via this preparation method by using needles with different calibers (Figure 2D). The particle size of the s-TS matrix can be flexibly determined according to the actual needs. By testing the particle size prepared by H_4_G_8_ and H_4_G_12_ polymer concentrations, it can be seen that the polymer concentration has little effect on the particle size. Particle stacking resulted in stress on the hydrogel beads at the bottom layer. Excessive stress can lead to the collapse of the stacking structure at the bottom layer, affect the gas pore distribution at the bottom layer, and hinder plant growth. The maximum load that the hydrogel particles at the bottom layer can withstand was determined by squeezing and injecting the s-TS in the syringe to simulate the particle stacking process, as shown in Figure 2E (Figure S2A,B). Both the polymer and the magnesium chloride concentrations could increase the maximum compression stress that the matrix could withstand. While keeping the thickener content unchanged, the maximum compression stress notably increased with the increasing content of the gelling agent because the magnesium chloride ions cross-linked with the gelling agent. Based on the permeability performance and bearing capacity of s-TS, we determined the size range of the rhizosphere observation box (50 mm × 20 mm × 100 mm for length, width, and height) according to the root length requirement of the plant, and then selected the corresponding concentration range of permeability and maximum compressive stress. To account for the pot diameter employed in this experiment, we prioritized selecting concentration ratios with higher transmittance. As a result, H_4_G_8_ 0.01 Mg hydrogel beads were chosen as the cultivation substrate for this study. During the cultivation, we need to consider that the hydrogel will be affected by water retention and it is difficult to maintain a full state for a long time. Therefore, we set up a more suitable gas cultivation environment (8 h/16 h dark/light environment, 99% humidity, room temperature) for the hydrogel substrate, under which we recorded the net weight of H_4_G_8_ 0.01 Mg hydrogel beads with and without plants for a long time. The water content of the hydrogel substrate can be kept stable for a long time, as shown in Figure 2F.

**Figure 3:**
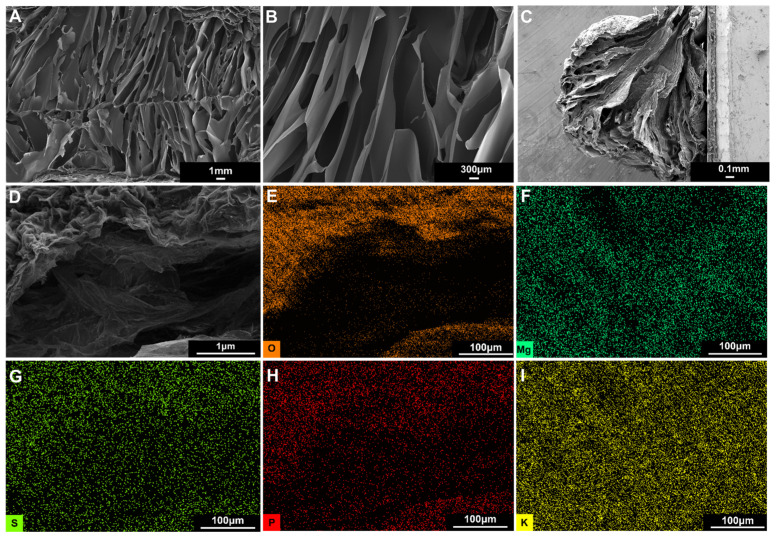
(**A**,**B**) Microporous three-dimensional network structure of H_4_G_8_ 0.01 Mg (1 × MS) hydrogels formed by ionic cross-linking. (**C**) Cross-sectional morphology of hydrogel particles. (**D**) Cross-sectional morphology of the interface of hydrogel particles. (**E**–**I**) Elemental distribution at the inner and outer interfaces of the H_4_G_8_ 0.01 Mg (1 × MS) spheres, corresponding to oxygen, magnesium, sulfur, phosphorus, and potassium, respectively.

Hydrogels can provide a sustained release of nutrients and water for root systems and supply the nutrients required for plant growth due to their high water content and salt tolerance. We observed the three-dimensional network structure of the H_4_G_8_ 0.01 Mg (1 × MS) hydrogel retained by freeze-drying through scanning electron microscopy, as shown in Figure 3A,B, which shows that the H_4_G_8_ 0.01 Mg (1 × MS) hydrogel has a rod-shaped porous structure inside, and the pore size reaches 1 mm, because the spiral structure of the freezing gel has strong rigidity and easily forms large pores after cross-linking. The H_4_G_8_ 0.01Mg (1 × MS) hydrogel beads were then freeze-dried, and the volume of the hydrogel beads shrank after drying (see Figure S2A,B), which may be caused by the different degrees of ion cross-linking between the inner and outer layers when the outer layer first forms a dense layer during vacuum freeze-drying and then collapses and shrinks inward. Figure 3C,D shows the cross-sectional morphology of the spherical s-TS particles after freeze-drying. It can be seen that the spherical structure shrinks as a whole, the surface is dense, and a slightly collapsed porous structure can be observed inside. We analyzed the element distribution at the interface between the inner and outer surfaces of the H_4_G_8_ 0.01 Mg (1 × MS) hydrogel beads and found that the polymer backbone oxygen, cross-linker magnesium, and nutrients (such as phosphorus, sulfur, potassium, and calcium, as seen in Figure S4) are uniformly distributed at the interface between the inner and outer surfaces of the hydrogel (Figure 3E–H), indicating that the hydrogel can provide nutrients for plant root systems.

**Figure 4:**
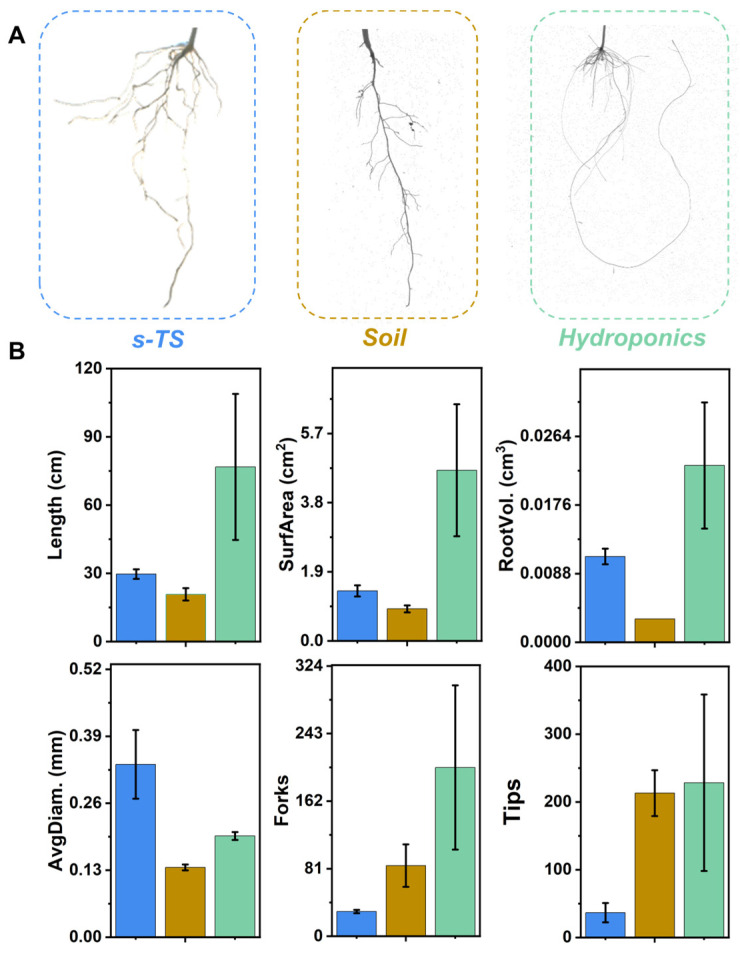
(**A**) High-resolution images of canola root systems after 60 d growth in H_4_G_8_ 0.01 Mg (1 × MS) hydrogel-based transparent soil (left), nutrient solution (middle), and soil (right) obtained by scanning with a flatbed scanner. (**B**) Statistical data on root length (Total Length), root diameter (AvgDiam), root surface area (SurfArea), root volume (Root Volume), number of forks (Forks), and number of tips (Tips) of root systems in the three environments, analyzed based on the images obtained from scanning. Error bars in the root phenotypic traits represent SD (*n* = 3).

**Figure 5:**
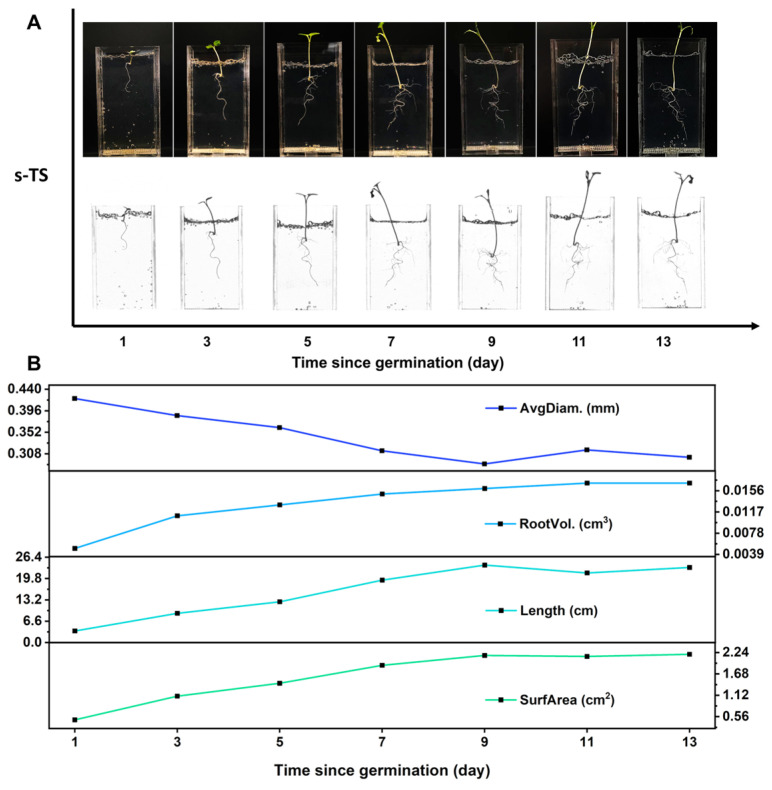
Dynamic growth process of B. napus in gel matrix. (**A**) Bright-field photographs of B. napus roots in H_4_G_8_ 0.01 Mg (1 × MS) (Mg^2+^ concentration: 0.01 mol·L^−1^, polymer: 1.2 wt.%) cell for 13 d after transplantation, scanned after filling the gap with 1 × MS nutrient solution. (**B**) Trends over time in the four root phenotypes in H_4_G_8_ 0.01 Mg (1 × MS) included average root diameter (AvgDiam.), root volume (RootVol.), root length (Length), and root surface area (SurfArea).

To compare the differences in root phenotypic traits among three substrates (H_4_G_8_ 0.01 Mg (1 × MS) hydrogel-based transparent soil, soil and nutrient solution) for rapeseed roots, we quantified six root phenotypic traits (Length, AvgDiam., SurfArea, RootVol, Forks, and Tips) for comparative analysis. First, we planted oilseed rape seedlings (3d) in H_4_G_8_ 0.01 Mg (1 × MS), 1 × MS nutrient solution and soil. Shown in Figure 4A are pictures of the oilseed rape root systems grown in H_4_G_8_ 0.01 Mg (1 × MS), soil, and nutrient solution for 60 days. It can be observed that the root systems in soil and gel have visible branches and nodules, and the lateral roots are arranged side by side on both sides of the primary root, while the lateral roots in the nutrient solution are concentrated at the root diameter of the primary root, of which the root diameter in soil and H_4_G_8_ 0.01 Mg (1 × MS) is significantly thicker than that in nutrient solution. Intuitively, the root system structures in H_4_G_8_ 0.01 Mg (1 × MS) and soil have higher similarity and are markedly different from the root structure in the nutrient solution. To further highlight this variability, roots were scanned and imaged using a flatbed scanner for quantitative analysis, and the results are shown in Figure 4B. These results are presented in the following order: root length (Total Length), root diameter (AvgDiam), root surface area (SurfArea), root volume (Root Volume), forks (Fork), and tips (Tips) for rapeseed roots in the three substrates. First, the root length and root surface area in H_4_G_8_ 0.01 Mg (1 × MS) were similar to those of the soil roots, whereas the data obtained from water culture were more than three times those of H_4_G_8_ 0.01 Mg (1 × MS) and soil. Second, the root volume and number of forks in s-TS and soil were relatively close, differing significantly from the results from water culture. The number of root tips in H_4_G_8_ 0.01 Mg (1 × MS) was smaller, while the number of root tips and the average root diameter in soil and water culture were similar. A higher average root diameter is more favorable for plant growth. When the nutrients around the roots are reduced, the root system of a plant can more easily explore nutrients in the loose accumulation system, and a sufficient water–oxygen environment helps the roots to exchange gases, reduce the retention of carbon dioxide, and prevent the accumulation of carbon dioxide in the rhizosphere, forming an acidic environment. A healthier root structure provides better support and a more stable nutrient supply for plant growth. Quantification of these six characteristics shows similar results to Figure 4A (Figure S5).

s-TS can supply moisture, nutrients, gas, and physical support for growing roots. It is kept in a drained, non-waterlogged condition during planting and a nutrient solution is added when imaging. On the one hand, this can supplement inorganic salts for s-TS; on the other hand, it helps reduce light loss from refraction during root imaging, thus achieving dynamic in situ root imaging. We moved 3-day-old rapeseed seedlings into the observation window of the H_4_G_8_ 0.01 Mg (1 × MS) we prepared and cultured them for 60 days, to obtain information about the dynamic growth process of rapeseed roots in the H_4_G_8_ 0.01 Mg (1 × MS) substrate. As shown in Figure 5A, from the first day to the thirteenth day after transplanting, high-definition color images and scans of the rapeseed root system in the observation window show that the rapeseed root system was simply a curled primary root on the first day. Subsequently, as the days after transplanting increased, the primary root gradually elongated and, starting on the third day, the mature zone gradually grew short lateral roots, which began to elongate as the days after transplanting increased. An analysis of each stage of root growth gave the four major characteristics of the roots (root diameter, root volume, root length, and root surface area), as shown in Figure 5B. Based on the data analysis, the average root diameter of rapeseed seedlings decreased with the increasing days after transplantation and was stable on the 9th day. However, the root volume, root length, and root surface area increased with the increasing days after transplantation and reached a plateau on the 9th day. Combined with the information seen in Figure 5A, this may be because the 3-day-old rapeseed seedlings had only one primary root with a thick diameter. Lateral roots started to emerge from the 3rd day and reached a stable root diameter by the 9th day. The elongation and increased number of lateral roots significantly contributed to the total root length, root volume, and root surface area. After the 9th day, the growth of the root system slowed down, and the data tended to be stable.

**Figure 6:**
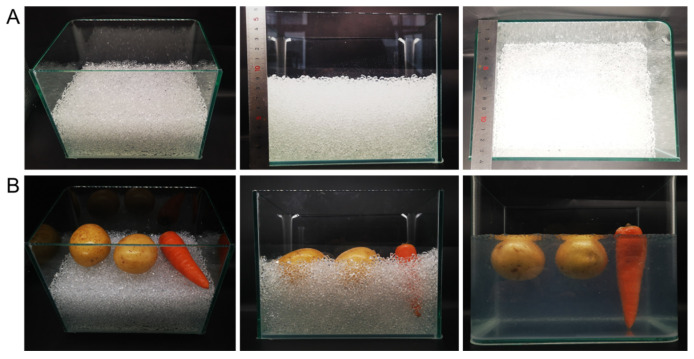
(**A**) Glass tank filled with swollen H_4_G_8_ 0.01 Mg (1 × MS), showing stereo image, front view, and top view, respectively, highlighting the self-standing height (90 mm) and width (135 mm) of the hydrogel. (**B**) Stereo image of s-TS surface populating with a potato and a carrot. Middle: load-bearing capability after large tubers were buried into s-TS. Right: clear visibility of large tubers from the observation window of H_4_G_8_ 0.01 Mg (1 × MS) after filling the gap.

In order to intuitively show the load capacity and transparency of hydrogel particles after stacking, we designed a glass cylinder (length × width × height: 200 mm × 140 mm × 160 mm), as shown in Figure 6. From left to right in Figure 6A are the three-dimensional diagram, front view, and top view of H_4_G_8_ 0.01 Mg (1 × MS) particles after self-stacking (stacking height 90 mm). After dry stacking, the particles will form a large number of channels connected to the atmosphere, which is convenient for root gas exchange. When the stacking height reaches 90 mm, it can be observed that the glass cylinder has the same color from top to bottom, and there is no visible difference in light transmission, indicating that the H_4_G_8_ 0.01 Mg (1 × MS) particles can completely retain the stacking channels at this time and have good bearing capacity. Figure 6B shows the overall display of introducing large rhizomes (196 g of potato and 68 g of carrot). The middle shows the bearing capacity after the introduction of foreign objects, and the right shows the transparency when the root system of the large rhizome is completely filled in the gaps between the particles. The results show that the hydroxyethyl cellulose hydrogel H_4_G_8_ 0.01 Mg (1 × MS) hydrogel-based transparent soil has good bearing capacity and transparency, which is not only conducive to the self-supporting growth of plant roots, but can also realize ultra-clear visual observation.

## 3. Materials and Methods

Materials. Low acyl gellan gum (La GG, average molecular weight ~275 kDa, with a de-acylation degree of ~98%) was purchased from Shanghai Yuanye Biotechnology Co. Ltd., Shanghai, China, hydroxyethyl cellulose (HEC, RG, 1500~2500 mPa.s) was purchased from Adamas-beta, magnesium chloride hexahydrate (MgCl_2_·6H_2_O, AR, ≥98.0%) was purchased from General-reagent, and MS medium (without agar and sucrose) was purchased from Compass Bio, Foshan, China, and all solutions were configured with deionized water. The oilseed rapeseeds used (B.Napus, hybrid, No. GPD Oilseed Rape (2018) 510112) were purchased from Sichuan Fule Seed Industry Co., Mianyang, China.

Preparation of s-TS. First, hydroxyethyl cellulose (HEC, 0.4 g) was gradually added to deionized water (100 mL) under magnetic stirring at room temperature to disperse the HEC well. Then, different amounts of low acyl gellan gum (0.4–0.8 g) were added slowly and dispersed. The mixture was stirred at 600 rpm for 2 h at room temperature. Subsequently, the mixed solution, which gradually transformed from turbid to semitransparent, was heated at 80 °C in a water bath for 3 h. The resultant homogeneous and transparent polymer solution was designated as the H_4_G_X_ (where x = 0.4–1.2 g) polymer solution. The obtained H_4_G_X_ polymer solution was allowed to stand in a water bath at 25–40 °C until the mixed solution reached thermal equilibrium. Six hydrated MgCl_2_·6H_2_O salt solutions with concentrations ranging from 0.01 to 0.09 mol·L^−1^ were prepared to act as a precipitation bath. Each 200 mL polymer solution was matched with 1 L of MgCl_2_ precipitation bath, the polymer solution dropwise falling into the coagulation bath to produce H_4_G_X_ mMg hydrogel microspheres (m = 0.01–0.09 mol·L^−1^). We collectively referred to them as s-TS. A facile hydrogel microsphere self-assembly device was constructed using metal spinnerets of different models (flow rate of 2.6 mL·min^−1^), a peristaltic pump, 30 cm of (2.1 mm inner diameter) infusion tube, two-jaw clamps, an iron stand, and a Luer head adapter. The polymer solution dripping into the coagulation bath rapidly formed gel microspheres. The microspheres were soaked in the coagulation bath for 2 h before being designated as s-TS.

Preparation of H_4_G_8_ 0.01 mg (1 × MS) hydrogel beads. Soak 500 mL of H_4_G_8_ 0.01 Mg (1 × MS) hydrogel beads in 1 L of 1 × MS nutrient solution for 6 h, remove H_4_G_8_ 0.01 Mg (1 × MS) hydrogel beads, and wait for backup.

Characterization of Transparent Soil. The EDX mapping is recorded on the SEM. The morphology, structure, and elemental distribution of the hydrogels after gold spraying were scanned with a Field Emission Scanning Electron Microscope (SEM, SU8010, Hitachi, Japan). Fourier transform infrared spectra (FTIR, Nicolet iS50, Benton, ME, USA) were collected using a universal attenuated total reflectance sampling accessory in the frequency range of 525–4000 cm^−1^.

UV Tests. The UV–vis absorption spectra were collected using a SHIMADZU UV 1900i (Kyoto, Japan) spectrophotometer (250–1100 nm). S-TS particles were stacked in a quartz cuvette of unit width in a close arrangement, and the interstices of the particles were filled with water, so that the light passing through the beads could minimize the loss caused by refraction and scattering within the optical range. The transmittance of the light of different wavelengths per unit length was measured within the optical range. It would be more reasonable to reproduce the conditions which actual observations of roots occur under, and calculate the maximum optical path that can be achieved when the transmittance is reduced to 10% according to the transmittance at 1080 nm, which is the maximum visualization width.

Mechanical Tests. The mechanical tests were carried on an electronic universal testing machine (1KN/UTM2103, Suns, Shenzhen, China) with the compressing rate of 5 mm·min^−1^.

In order to simulate the maximum pressure that the particles can withstand when compressed to collapse in a semi-closed system, a fixed volume (2 mL) of hydrogel beads was loaded into a syringe and compressed by a universal testing machine to obtain their maximum pressure, and the corresponding stackable depth was calculated by the maximum pressure. The specific process is as follows:

$$\frac{N_{1}}{S_{1}}=\frac{N_{2}}{S_{2}}$$

*N*_1_ is the gravity of loading weights; *S*_1_ is the area of the syringe plug bottom surface; *N*_2_ is the gravity of the above beads; *S*_2_ is the area of the interface between the above layer and the bottom layer beads.

$$N_{1}=mg$$

where m is the mass of loading weight and g is the uniform acceleration.

$$S_{1}=\frac{V_{1}}{h_{1}}$$

where *V*_1_ is the volume of above beads (10 mL); *h*_1_ is the height of the syringe (6.25 cm).

$$S_{2}=\frac{V_{2}}{h}=\frac{M}{\rho h}=\frac{N_{2}}{\rho gh}$$

where *V*_2_ is the volume of above beads; *h* is the height of above beads; *ρ* is the density of TS beads; Mis the mass of above beads.

Therefore,

$$h=\frac{mh_{1}}{\rho V_{1}}$$

Water Content Measurement. The water retention of the hydrogel was tested under set cultivation conditions, and it was found that the substrate was able to keep the system stable without decomposing during cultivation. The water content of the samples was measured over time by weighing three sets of samples of similar mass, recording the mass of the containers as well as the starting mass of the samples, and subsequently recording the total weight and subtracting the mass of the containers every 48 h:

$$W_{c}(\%)=\frac{M_{s}-M_{d}}{M_{s}}100\%$$

where *M_s_* and *M_d_* are the gel weights before and after 48 h, respectively.

Average Diameter Measurements. Five different sizes of particles (including diameter sizes of 0.9 mm, 1.8 mm, 2.2 mm, 3 mm, 3.4 mm) were made with needles of different pore sizes, and the changes in the diameter of the prepared gel balls were measured with the change in the pore size of the needle. The average diameter size of the gel balls was obtained by a set of five gel beads measured side by side with the length averaged over three consecutive measurements.

Root Trait Measurements. Root phenotypic imaging analysis was conducted using a root analyzer (800 ppi/HED-WinRHIZO, Huoerde, Shandong, China) to compare root traits across different substrates: nutrient solution, soil, and a transparent gel developed in this study. The process involved several steps: (1) Seed treatment: Oilseed rape and Arabidopsis thaliana seeds were germinated on moistened germination paper. After 36 h, seedlings with similar hull breaking were hydroponically grown for two days, then transplanted into different environments. (2) Transplanting seedlings: In both soil and the experimental transparent gel substrate, a 4 cm deep hole was made to plant the seedlings. Three seedlings per tube were covered with soil or gel to tightly enclose the substrate around the roots. This process was replicated over three times for each experimental group, H_4_G_8_ 0.01 Mg hydrogel was soaked in nutrient solution every three days for 6 h, and then emptied (room temperature, 99% air humidity, 16 h light/8 h dark cycle). (3) Sampling: After uniform growth, the soil and substrate were carefully removed, and the roots were rinsed with water to ensure complete dispersion without damage. (4) Testing: The root systems were scanned and imaged using the root scanner.

## 4. Conclusions

In this paper, by employing a mixture of stable, colorless, and water-soluble non-ionic polymer hydroxyethyl cellulose and ion-sensitive low acyl gellan gum, we fabricated a super-transparent soil (s-TS) with a transmittance of up to 98.45%, which broadens the width of the hydrogel-based observation window for transparent soil. When the transmittance is kept at 10%, the width of the system can reach 147.4 cm. Moreover, the transmittance of s-TS of all proportions is maintained above 89.5% within the concentration range. The maximum bearing capacity of s-TS can reach 186 cm, allowing the maximum height of the observation window to be 477 kPa, significantly broadening the height of the hydrogel-based transparent soil observation window. SEM observation reveals that s-TS has a large porous structure with uniform ion distribution, which is beneficial to plant roots absorbing moisture and nutrients from hydrogel beads. Rapeseed was planted in s-TS and compared with nutrient solution and real soil. The roots of rapeseed grown in hydrogel are closer to those grown in real soil, which demonstrates its potential in plant phenotype analysis. Currently, the studied hydrogel-based s-TS is an improvement based on natural culture medium. Although it can greatly optimize the performance and reduce the cost in material design, it inevitably affects the rhizosphere microbiota. Next, through the surface treatment of gel particles or the design of base materials, we expect to break free from the limitations of aseptic culture and realize the study of plant rhizosphere microbial communities, which will bring new directions to the hydrogel-based s-TS.

## Data Availability

Data are contained within the article and Supplementary Materials.

## Supplementary Materials

The following supporting information can be downloaded at: https://www.mdpi.com/article/10.3390/molecules29112677/s1.
