# Supplementary material for: Super-Transparent Soil for In Situ Observation of Root Phenotypes
Source: Molecules. 2024 Jun 5;29(11):2677. doi: 10.3390/molecules29112677 (PMC11173578; doi:10.3390/molecules29112677)
Supplement: Supplementary file 1 [file molecules-29-02677-s001.zip › molecules-2985752-supplementary.pdf]

SUPPORTING INFORMATION

Super-transparent Soil for In-situ Observation of Root Pheno-types

Jinchun Xie [1], Qiye Wu [1], Liping Feng [2], Junfu Li [1], Yingjie Zhou [1], Guozhang Wu [2,*] and Yongjun Men [1,*]

1. State Key Laboratory for Modification of Chemical Fibers and Polymer Materials, College of Materials Science and Engineering, Donghua University, Shanghai 201620, China; e-mail: y.men@dhu.edu.cn

2. Joint Center for Single Cell Biology, School of Agriculture and Biology, Shanghai Jiao Tong University, 800 Dongchuan Road, 200240 Shanghai, China; e-mail: gzwu@sjtu.edu.cn

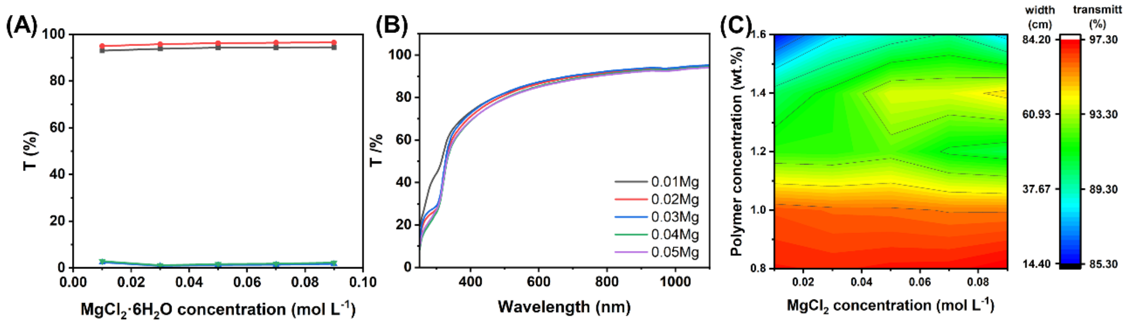

Figure S1. (A) s-TS gel beads (H_4G_8nMg, n=0.01 ~ 0.09 mol·L^{-1}) in saturated state (upper) and unsaturated state (lower) at 800 nm (black, blue) and 1080 nm (red, green) UV transmittance. (B) Transmittance curves of s-TS gel beads (H_4G_8) treated with different Mg ion concentrations (0.01-0.05 mol·L^{-1}) in the wavelength range of 250 - 1100 nm. (C) Transmittance and maximum visualization width of s-TS at 800 nm.

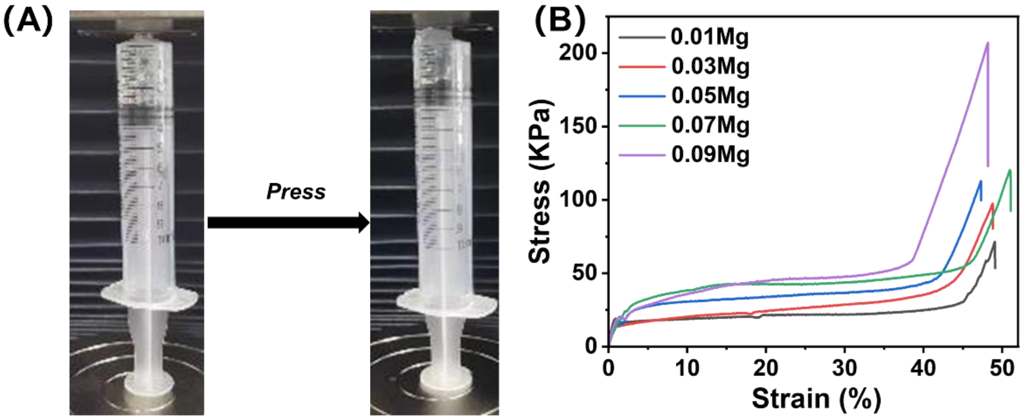

Figure S2. (A) Compression experiment performed by stacking s-TS beads inside a syringe. (B) Compression curve of TS beads (H_4G_8nMg, n=0.01 ~ 0.09mol·L^{-1}).

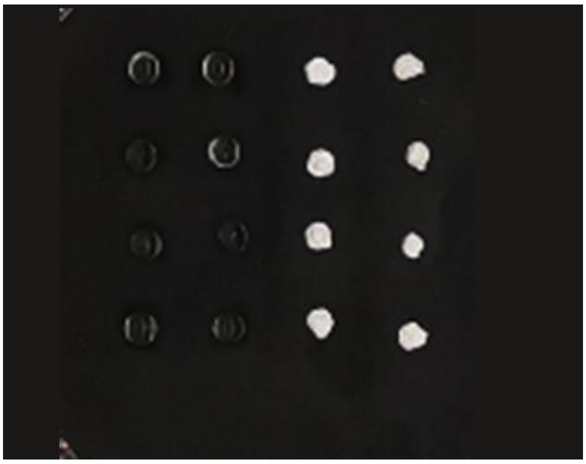

Figure S3. Pictures of s-TS beads before (left) and after (right) lyophilization.

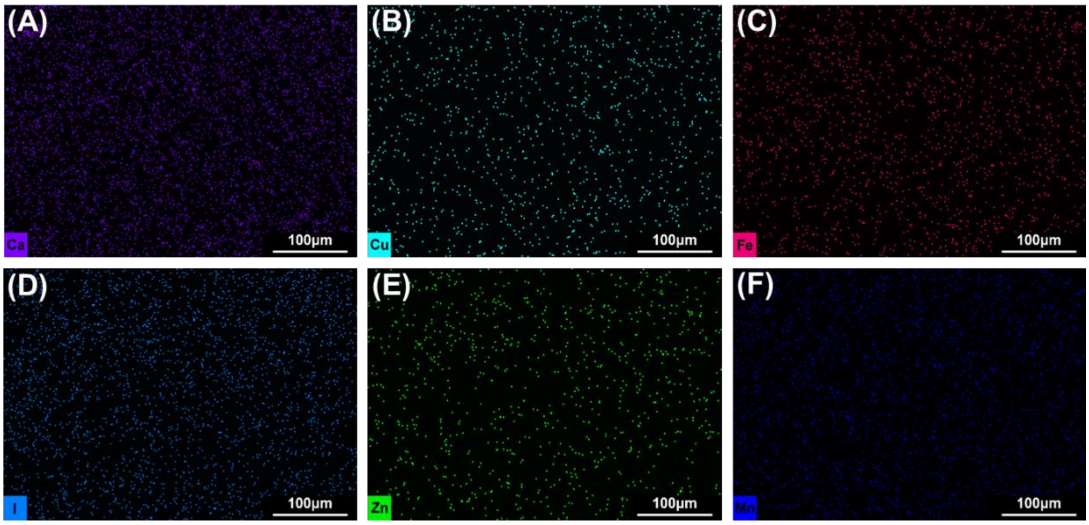

Figure S4. (A-F) EDX images at the internal and external interfaces of TS gel beads.

X-ray diffraction (XRD) patterns were obtained over 2θ ranging from 10 to 60°at a scanning rate of 5 °·min^{-1} on a Powder X-ray diffractometer (Bruker D2 Phaser/D2 phaser, Germany).

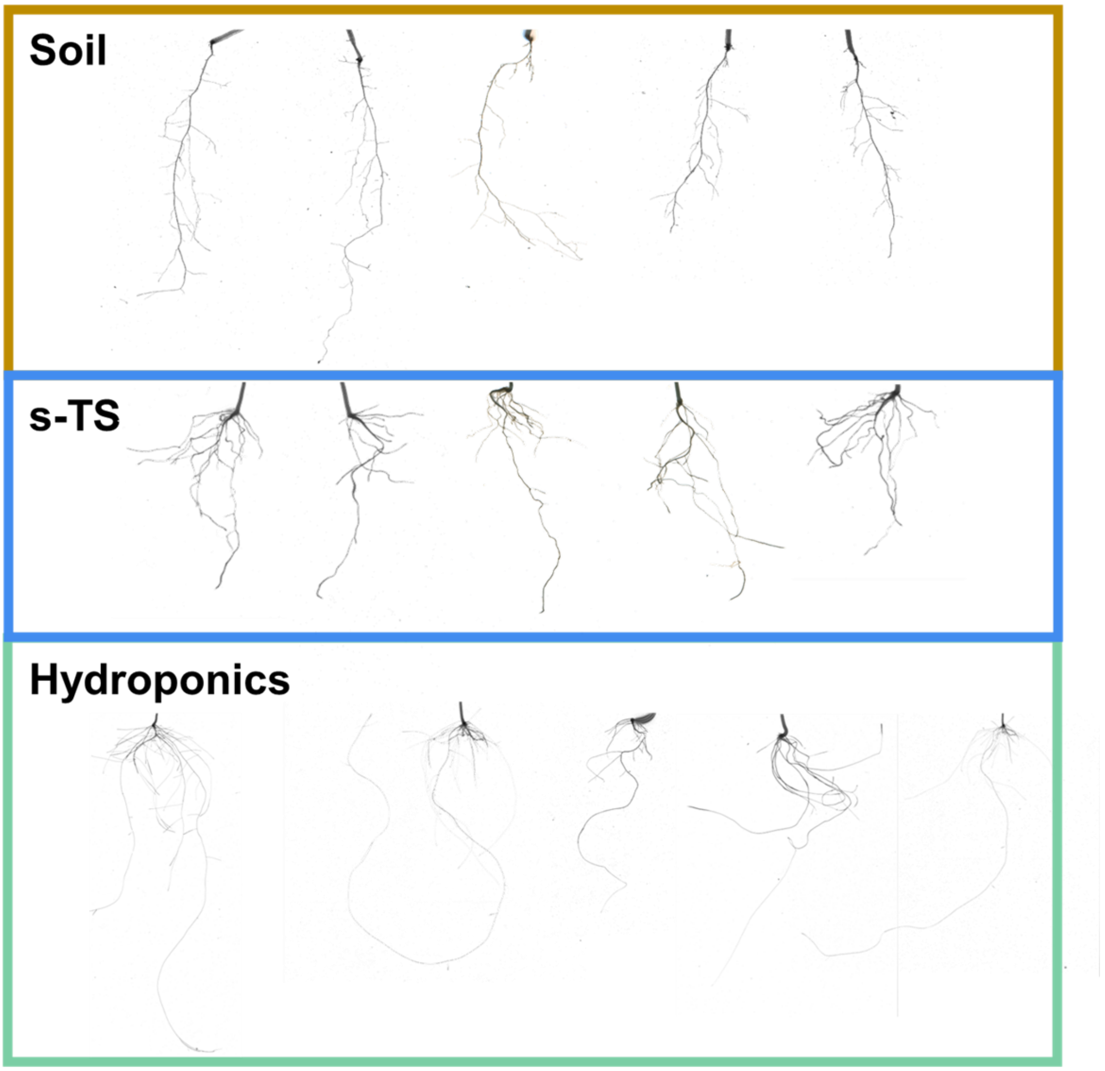

Figure S5. Root phenotype imaging images of rapeseed roots in soil, s-TS, and hydroponics.
